# 2474. Emergence of *Candida auris* in Michigan

**DOI:** 10.1093/ofid/ofad500.2092

**Published:** 2023-11-27

**Authors:** Sara E McNamara, Brenda M Brennan, Niki Mach, Nicole McGuire, Margaret Sturgis, Jane E Rogers, Anurag Malani, jason M Pogue

**Affiliations:** State of Michigan, Lansing, Michigan; Michigan Department of Health and Human Services, Lansing, Michigan; Michigan Department of Health and Human Services, Lansing, Michigan; Michigan Department of Health and Human Services, Lansing, Michigan; Michigan Department of Health and Human Services, Lansing, Michigan; Michigan Department of Health and Human Services, Lansing, Michigan; Trinity Health Michigan, Ann Arbor, Michigan; University of Michigan, College of Pharmacy, Ann Arbor, Michigan

## Abstract

**Background:**

*Candida auris* is an emerging multidrug-resistant fungal pathogen associated with high rates of morbidity and mortality, and outbreaks in healthcare facilities (HCF). In May 2021, Michigan detected its first case of *C. auris* and has since seen an increase in *C. auris* cases. We aimed to characterize the epidemiology of the emergence of *C. auris* in Michigan and subsequent regional public health containment response activities.

**Methods:**

Since 2018, cases of *C. auris* from clinical or colonization screening specimens are reportable to public health in Michigan. Epidemiologic information collected on each case included clinical status and HCF exposures. Contact investigations and colonization screenings were conducted to identify potential healthcare contact exposures. Facilities with confirmed cases were set up with Infection Control and Assessment Response (ICAR) visits to identify and address infection prevention and control (IPC) practice gaps. Action plans outlining all recommendations and resources were provided.

**Results:**

From May 2021 – March 2023, 224 cases of *C. auris* have been identified in Southeast Michigan. 159 were from screening specimens and 65 from clinical specimens (25 of which were collected from patients previously known to be colonized). Cases were detected at 28 HCFs across 7 local health jurisdictions in Southeast MI, most frequently at long-term acute care hospitals (LTACHs; 103 cases), followed by acute care hospitals (98 cases). 2663 response-driven colonization screenings of healthcare contacts were collected at 56 HCFs, with the highest percent positive in LTACHs (9.3%). 32 ICAR visits were conducted which identified areas for improvement in IPC practices, personal protective equipment use, and cleaning and disinfection practices.

**Figure:**
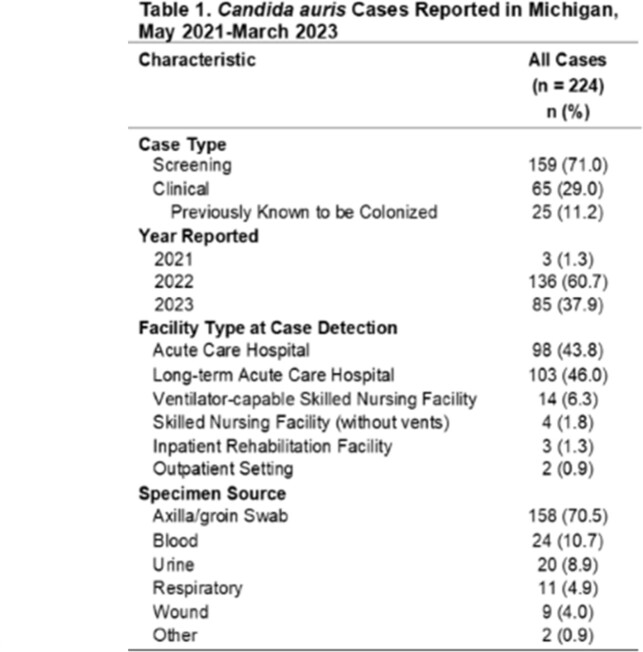

**Figure:**
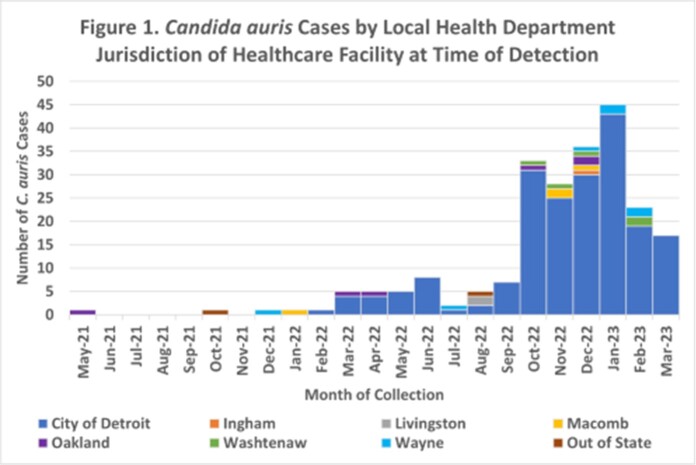

**Figure:**
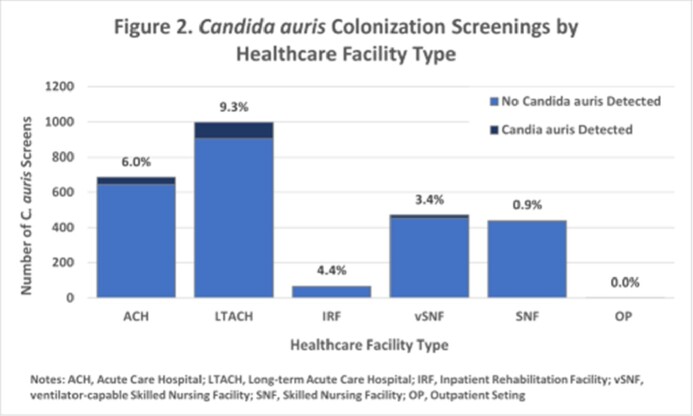

**Conclusion:**

*C. auris* has recently emerged in Southeast Michigan HCFs with a 44-fold increase in cases within the second year. Vulnerable patient populations were at highest risk including those in high acuity care in a hospital or long-term care setting. Enhanced surveillance, early detection, and a robust public health containment response, including colonization screenings and assessments of IPC practices are essential to help mitigate future outbreaks and further spread of *C. auris* in MI HCFs.

**Disclosures:**

**jason M. Pogue, PharmD**, AbbVie: Advisor/Consultant|Entasis: Advisor/Consultant|Ferring: Advisor/Consultant|GSK: Advisor/Consultant|Merck: Advisor/Consultant|Merck: Grant/Research Support|Qpex: Advisor/Consultant|Shionogi: Advisor/Consultant

